# Study on the Common Molecular Mechanism of Metabolic Acidosis and Myocardial Damage Complicated by Neonatal Pneumonia

**DOI:** 10.3390/metabo13111118

**Published:** 2023-10-30

**Authors:** Yifei Zhan, Huaiyan Wang, Zeying Wu, Zhongda Zeng

**Affiliations:** 1 College of Environmental and Chemical Engineering, Dalian University, Dalian 116622, China; zhanyifei@chemdatasolution.com; 2 Department of Neonatology, Changzhou Medical Center, Changzhou Maternity and Child Health Care Hospital, Nanjing Medical University, Changzhou 213000, China; huaiyanwang@njmu.edu.cn; 3 State Key Laboratory of Analytical Chemistry for Life Science, School of Chemistry & Chemical Engineering, Nanjing University, Nanjing 210023, China; 4 School of Chemical Engineering and Material Sciences, Changzhou Institute of Technology, Changzhou 213032, China

**Keywords:** UPLC–HRMS-based metabolomics, chemometrics, pneumonia, metabolic acidosis, myocardial damage

## Abstract

Pneumonia is a common clinical disease in the neonatal period and poses a serious risk to infant health. Therefore, the understanding of molecular mechanisms is of great importance for the development of methods for the rapid and accurate identification, classification and staging, and even disease diagnosis and therapy of pneumonia. In this study, a nontargeted metabonomic method was developed and applied for the analysis of serum samples collected from 20 cases in the pneumonia control group (PN) and 20 and 10 cases of pneumonia patients with metabolic acidosis (MA) and myocardial damage (MD), respectively, with the help of ultrahigh-performance liquid chromatography–high-resolution mass spectrometry (UPLC–HRMS). The results showed that compared with the pneumonia group, 23 and 21 differential metabolites were identified in pneumonia with two complications. They showed high sensitivity and specificity, with the area under the curve (ROC) of the receiver operating characteristic curve (ROC) larger than 0.7 for each differential molecule. There were 14 metabolites and three metabolic pathways of sphingolipid metabolism, porphyrin and chlorophyll metabolism, and glycerophospholipid metabolism existing in both groups of PN and MA, and PN and MD, all involving significant changes in pathways closely related to amino acid metabolism disorders, abnormal cell apoptosis, and inflammatory responses. These findings of molecular mechanisms should help a lot to fully understand and even treat the complications of pneumonia in infants.

## 1. Introduction

Neonatal pneumonia is a common disease in very young infants. It is the leading cause of death in high under-5 mortality rate (U5MR) countries [1] and the major single killer of children outside the neonatal period [2]. Atypical clinical manifestations and the rapid onset and progress of neonatal pneumonia often result in associated complications such as respiratory failure, which presents a serious threat to children’s health. Thus, early, accurate diagnosis and timely and effective treatment are particularly important.

However, due to the various causes of pneumonia infections, the symptoms of pneumonia are diverse and difficult to diagnose [3,4,5]. X-ray, CT examinations, and lung ultrasonography have been implemented for the diagnosis of pneumonia, but these methods have several disadvantages, such as the limited accuracy of imaging diagnosis, inconveniences, and high costs, resulting in a great challenge in current clinical practice [6,7]. Furthermore, there is also a lack of clinically reliable means for the prediction, classification, or effective screening of patients who require a higher level of care, making the appropriate triage of newborns with pneumonia rather problematic. Therefore, a thorough investigation of the molecular mechanisms of neonatal pneumonia is highly in demand, which will provide an in-depth understanding of the causes, identification and prevention approaches, and clinical management for patients.

Metabolomics, which bridges the gap between scientific interests and biological findings, is a strategy to understand and diagnose diseases, and reveal the mechanisms of disease occurrence and development. By obtaining the results of small molecule changes in metabolic phenotypes, it is possible to discover and explain the internal mechanisms that lead to diseases, and this is very important for early detection and treatment, leading to a decline in prevalence [8,9]. For example, Li et al. performed ultrahigh-performance liquid chromatography–tandem mass spectrometry analysis of metabolites in urine samples of healthy children and children with mycoplasma pneumoniae pneumonia in children (MPPC) [10]. In their study, acetyl phosphate and 2, 5-dioxovalerate were recognized for the first time as potential biomarkers for early diagnosis of MPPC. A similar approach was used by Del Borrello et al. to successfully reveal metabolic changes in community-acquired pneumonia (CAP) [11]. Three metabolites, sphingosine, lactate, and DHEA-S, were discovered to represent a panel of potential small molecule biomarkers for assessment of the severity of CAP [12]. The suitability of a new set of serum biomarkers, consisting of two proteins and three metabolites, for the identification of CAP and the recognition of severe pneumonia was proved by Wang et al. [13]. In these reports, the mechanisms were interpreted, and the development and evolution of diseases were explained based on the information obtained from the metabolomic analysis results.

The above studies have demonstrated the applicability of metabolomics for the pathogenic diagnosis of pneumonia and the assessment of pneumonia severity. However, the integrated and common molecular mechanisms of pneumonia with complications failed to be thoroughly investigated previously. Metabolomics-oriented research into the distinction between neonatal pneumonia and pneumonia complications is relatively rare, and clinical diagnosis, classification, and staging for pneumonia complications are also very limited.

In this work, a nontargeted metabolomics method using ultrahigh-performance liquid chromatography–high-resolution mass spectrometry (UPLC–HRMS) was established at first. The metabolites in serum samples collected from the control group suffering from pneumonia only (PN group), from patients suffering from pneumonia, metabolic acidosis, and other medical conditions except for myocardial damage (PN&MA group), and from patients suffering from pneumonia and myocardial damage (PN&MD group) were then fully investigated and compared with the PN group statistically. A comprehensive analysis of differences in the identified metabolites between PN, MA, and MD was performed for the discovery of key compounds involved in pneumonia complications. The common metabolite molecules were recognized and elucidated. The changes in these compounds and their metabolic pathways in neonatal pneumonia complications were further explored.

## 2. Materials and Methods

### 2.1. Sample Collection

This study was approved by the Ethics Committee of Changzhou Maternal and Child Health Hospital (CMCHH), Changzhou, China (approval number: 2019017). The blood samples of newborns were collected from December 2019 to September 2020 at CMCHH.

**Figure 1:**
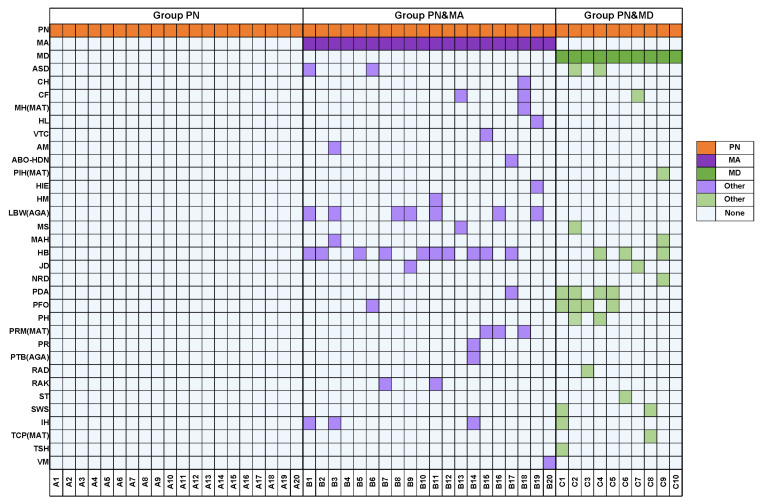
Clinical information of samples in groups PN, PN&MA, and PN&MD, respectively.

In this study, serum samples of 20 patients in PN group, 20 patients in PN&MA group, and 10 patients in PN&MD group were collected for nontargeted metabolomic analysis. The details of all the samples are shown in Figure 1. Most samples of patients with complications of MA or MD had other medical conditions. The details are given in the left column of the figure. The full names of all diseases with abbreviations are given in the Supplementary Material Table S1.

After collection, each blood sample was placed in a vacuum blood collection tube containing a procoagulant, left to stand for 1 h at room temperature, and centrifuged for 10 min at a speed of 3000 r/min. The supernatant was then placed in a centrifuge tube and stored in a refrigerator at −80 °C.

### 2.2. Materials

Methanol of chromatographic grade was purchased from Beijing Zhenxiang Company. All internal standards (IS) were obtained from the same company and prepared into a mixed standard solution with methanol. The final concentration of each standard in the mixed standard solution is given in Table 1.

### 2.3. Sample Preparation

The serum samples were prepared on ice. First, 50 μL sample was pipetted into a 1.5 mL centrifuge tube (Oxygen, Doral, FL, USA), 200 μL cold methanol was added, and it was shaken for 4 min at a speed of 1500 r/min on a Vortex Mixer T1 (Titan, West Springfield, MA, USA). The mixed solution was then left to stand for 10 min at a temperature of −20 °C prior to centrifugation for 15 min at 4 °C and 14,000 r/min. After this, 200 μL of supernatant was taken into a new centrifuge tube, while the remaining supernatant was used to prepare the quality control (QC) sample solution. The samples were then concentrated and stored at −40 °C. Prior to analysis, the lyophilized samples were redissolved in 100 μL methanol/water (80/20, *v*/*v*) solution, followed by shaking and centrifugation operations (1500 r/min). The supernatant was subject to analysis by UPLC–HRMS (Q-Exactive, Thermo Fisher, Waltham, MA, USA) in both positive and negative modes.

### 2.4. UPLC–HRMS Analysis

The UPLC–HRMS analysis was performed using a BEH C8 column (2.1 × 100 mm × 1.7 μm, Waters, Milford, MA, USA) in positive mode and an HSS T3 column (2.1 × 100 mm × 1.8 μm, Waters, USA) in negative mode. The flow rate was 0.35 mL/min on both columns. The mobile phases A and B were 0.1% formic acid in water and 0.1% methanol in acetonitrile, respectively. The gradient program started with 5% B and held for 1 min. Then, it was linearly changed to 100% B in 10 min and held for 2 min. The column temperature was set at 50 °C.

The ion source was operated with a spray voltage of 3.8 kV in positive mode and −3.0 kV in negative mode. The ion transfer capillary temperature was set at 320 °C. All the samples were analyzed in a nontargeted full scan acquisition mode from 70 to 1050 *m*/*z* at a resolution of 70,000. The MS^2^ measurement was performed in independent data acquisition (IDA)-based auto-MS^2^ mode, and the MS/MS fragments were acquired at a resolution of 17,500.

### 2.5. Data Processing and Statistical Analysis

The nontargeted raw data obtained from UPLC–HRMS analysis was first converted into mzML format using One-MAP/PTO software v2.8, which is freely available from www.5omics.com, accessed on 3 January 2023. It was further used to recognize and extract the MS characteristics of metabolites buried in the same sample and then to match the primary MS of the same substance in each sample to an Excel file for the generation of peak tables, which were the basis for the subsequent statistical analysis. The MS2 data were obtained from mgf files, in which the retention times and MS1 and MS2 information of MS features in peak tables were all included. Afterward, the peak lists in the table were preprocessed, such as filling in the missing values and sample normalization. The data quality was evaluated and calibrated by using One-MAP from www.5omics.com to improve the data quality. The peak table, together with the MS^2^ data matched, was then imported into .mgf format for annotation of each MS feature and discovery of differential metabolites. The annotation was attained according to the qualitative characteristics of each compound built in the database of One-MAP.

The recognition of differential metabolites between different clinical groups was achieved with the help of a combination of univariate and multivariate analysis. For univariate statistical analysis, a volcano plot with information combination of fold change (FC) > 1.5 and *p* < 0.05 was used to effectively identify those differential components with statistically significant changes amongst different groups. For multivariate analysis, partial least squares discriminant analysis (PLS-DA) was applied to model the main potential variables and then screen the characteristic ions/metabolites that differed in the group with the values of variable importance in the projection (VIP) larger than 1.0. The relative levels of metabolite differences in each group were expressed as upward and downward changes in the multiplicity of change to visually represent the variation in differential metabolites between the experimental and control groups. Metabolic enrichment analysis was also performed on the basis of the aforementioned results of annotation and differential discovery of metabolites. The pathways to which these metabolites corresponded were determined, and the clinical interpretation and potentials were explained afterward. Both the groups of PN and PN&MA and PN and PN&MD were fully investigated following the strategies and methods introduced above.

## 3. Results

### 3.1. Metabolic Profiling Analysis

**Figure 2:**
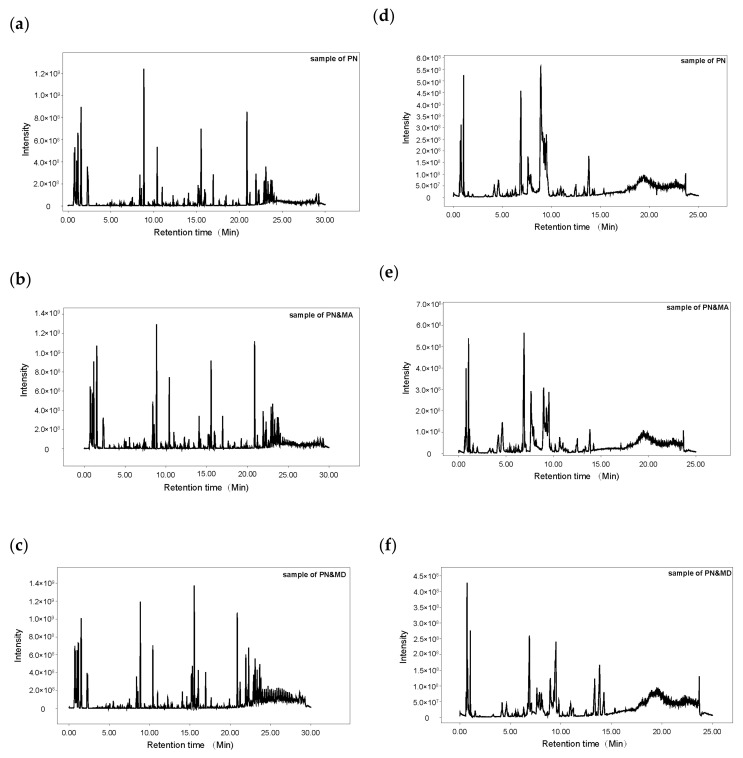
TICs of representative samples in groups PN (**a**,**d**), PN&MA (**b**,**e**), and PN&MD (**c**,**f**), respectively: (**a**–**c**) TICs measured in positive mode; (**d**–**f**) TICs measured in negative mode.

The typical total ion chromatograms (TICs) of serum samples in groups PN, PN&MA, and PN&MD obtained in positive mode are shown in Figure 2a–c, respectively. The corresponding TICs measured in negative mode are shown in Figure 2d–f, respectively. The horizontal axis in Figure 2 represents the retention time in minutes, while the vertical axis represents the peak intensity. It is obvious that all the samples were well separated under the experimental conditions described in Section 2.4 in both positive and negative modes.

**Figure 3:**
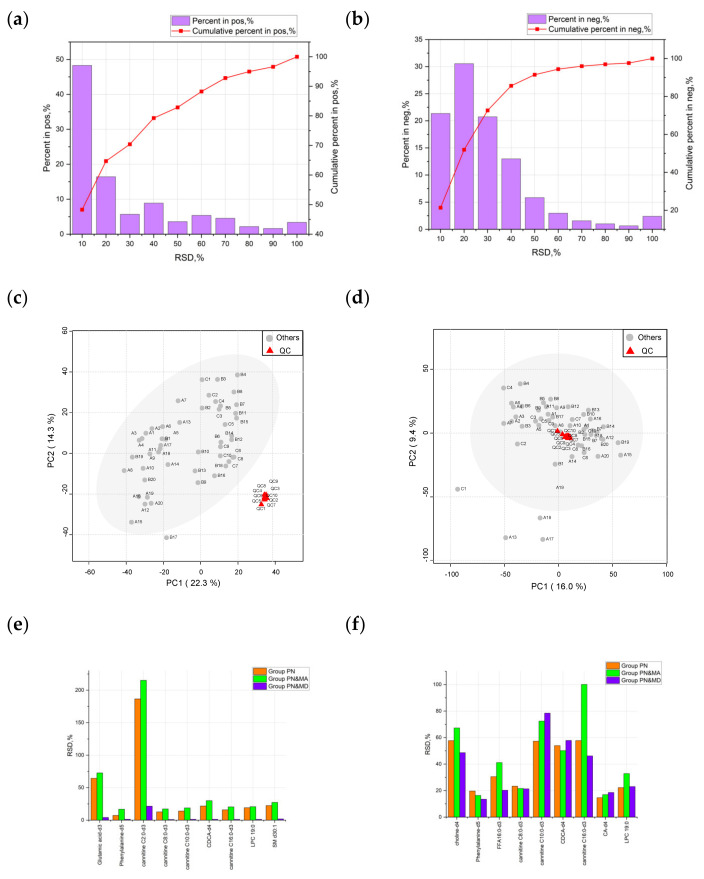
Quality evaluation based on QC samples and ISs. (**a**,**b**), (**c**,**d**), and (**e**,**f**) correspond to the results of RSD distribution of QC samples before data calibration, PCA evaluation results of QC samples, and RSD distribution of ISs in positive and negative mode, respectively.

It is well known that both QC samples and IS compounds can provide important information for data quality evaluation and analysis in metabolomics studies and can be further used for data calibration to improve data quality if required. Figure 3a,b illustrate the distribution of relative standard deviations (RSDs) of mass spectral features in QC samples before data calibration in positive and negative modes, respectively. It can be observed that the data quality of the QC samples was relatively satisfactory. The percentage of MS features in QC samples with RSDs lower than 30% was greater than 75%. On the other hand, the PCA results of QC samples showed good consistency in both positive and negative modes, as given in Figure 3c,d. This indicated a high quality of UPLC–HRMS measurement.

The IS compounds can be used to maintain overall data quality during the experimental process, although some of the ISs may not be stable during the whole procedure, due to which the data quality was reduced. The ISs with RSD values below 30% in positive mode accounted for 84.6%, as given in Figure 3e. Among all the ISs, the RSD of cannitine C2:0-d3 and glutamic acid-d3 were relatively high. In negative mode, as shown in Figure 3f, the percentage of ISs with RSDs lower than 30% was 44.4%, which was not as good as in positive mode. This was probably due to the suitability of these components to be ionized by positive ion sources. Combining the results of the data quality analysis as described above, the accuracy and reproducibility of the experimental analysis proved that the proposed approach was feasible for the present study.

### 3.2. Discovery of Differential Metabolites and Further Analysis of Metabolic Pathway

**Figure 4:**
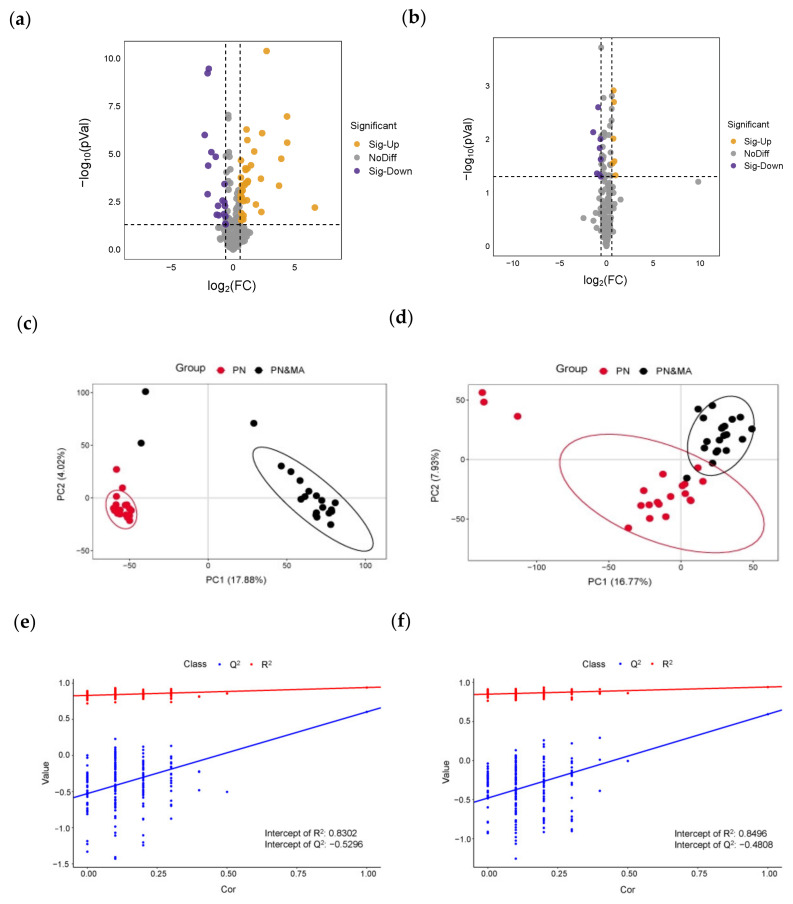
The results of univariate and multivariate analysis of PN and PN&MA groups. (**a**–**f**) were volcano plots, PLS-DA score plots, and permutation analysis obtained in positive and negative modes, respectively.

The processes for nontargeted metabolomics data analysis were previously introduced in Section 2.5. Due to the large variance in the interfering diseases in the samples, some of them exhibited outliers in the analysis, but a trend of separation was still observed. The annotation results are given in the Supplementary Material Table S2. In Figure 4a,b, univariate volcano plots of univariate analysis were obtained with statistical *p*-values lower than 0.05 and FCs larger than 1.5 in both positive and negative modes, respectively. In positive mode, the results of PLS-DA modeling revealed a significant difference between the PN&MA group and PN controls, as shown in Figure 4c. It is obvious that samples collected from healthy children and patients were discriminated with 100% accuracy. Similarly, a consistent pattern was also observed between these two groups in negative mode, as given in Figure 4d. With the help of univariate and multivariate analysis, a total of 23 metabolites were discovered to be significantly different in the PN and PN&MA groups. The permutation test plots with number of running time equal to 200 ensured the credibility of the proposed model, which are shown in Figure 4e,f. These results of the distribution of R^2^ and Q^2^ values indicated that the model was reliable and could be used for predictive analysis.

**Figure 5:**
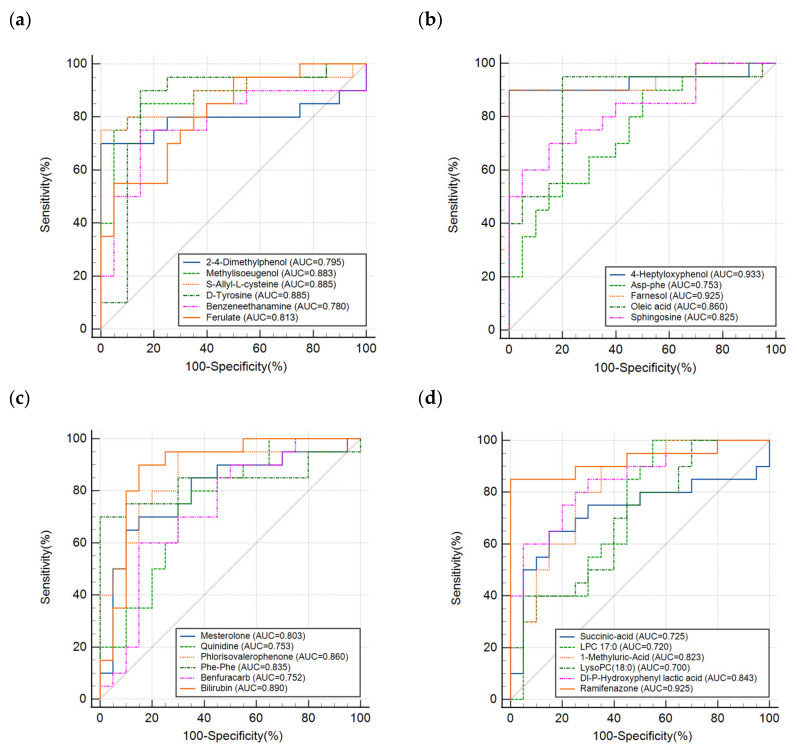
The results of ROC analysis of all the differential molecules obtained from PN and PN&MA groups. (**a**–**d**) correspond to the results obtained from different components, as shown in each figure.

In addition, the specificity and sensitivity of the ROC model evaluation in positive and negative modes reached 100% and 100%, and 95% and 80%, respectively. Figure 5 shows the ROC curves of each differential molecule, with AUC values above 0.7, for discrimination of PN and PN&MA groups, which indicated a high sensitivity and a good specificity of the 23 differential molecules for discrimination analysis.

**Figure 6:**
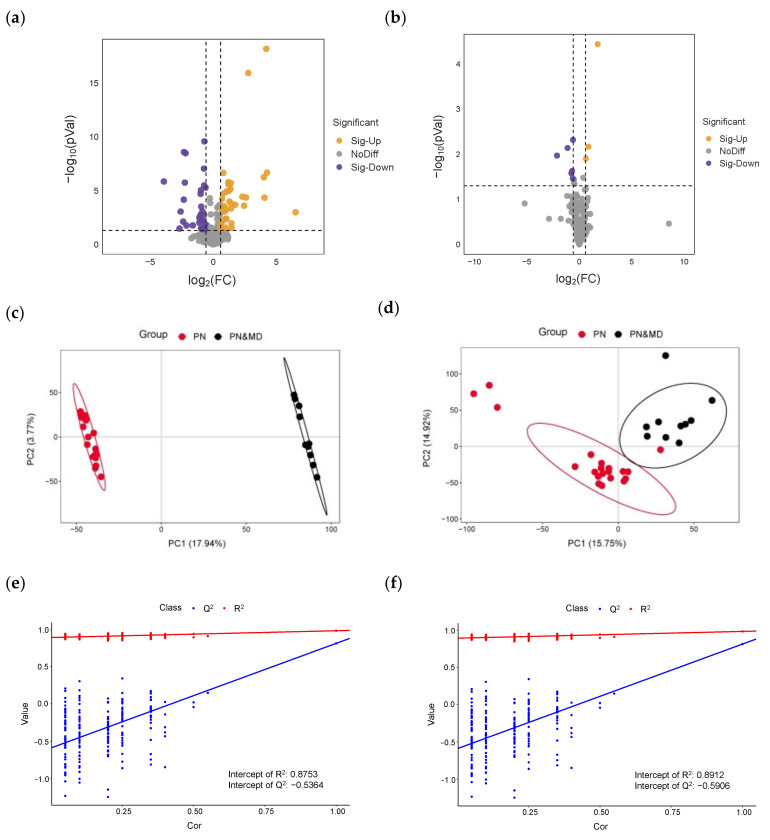
The results of univariate and multivariate analysis of PN and PN&MD groups. (**a**–**f**) are volcano plots, PLS-DA score plots, and permutation analysis obtained in positive and negative modes, respectively.

**Figure 7:**
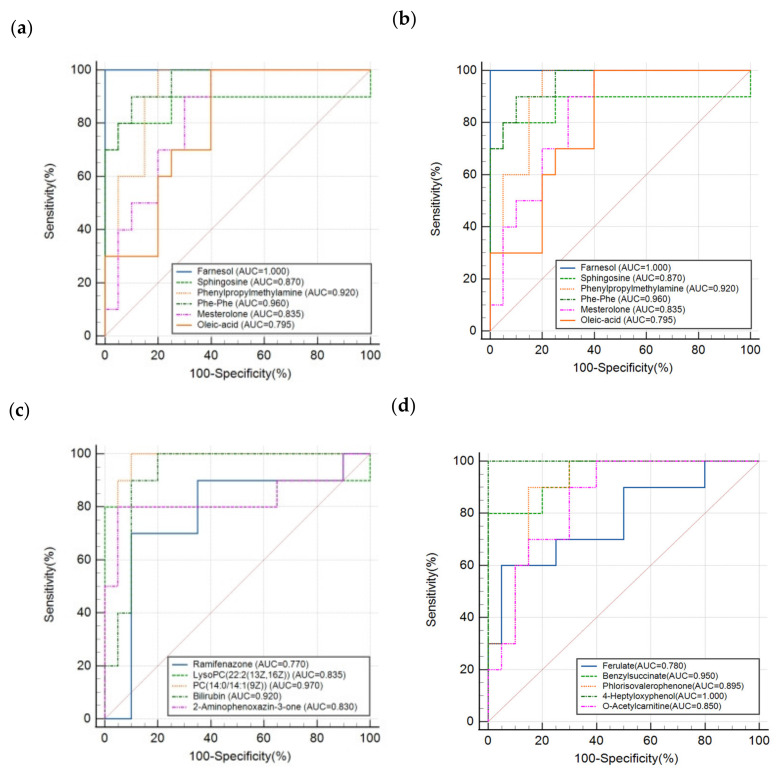
The results of ROC analysis of all the differential molecules obtained from PN and PN&MD groups. (**a**–**d**) correspond to the results obtained from different components, as shown in each figure.

Figure 6a,b are volcano plots in positive and negative modes obtained from univariate statistical analysis with *p*-values lower than 0.05 and FCs larger than 1.5. In terms of the processes for data analysis introduced above, the PLS-DA models of PN and PN&MD groups indicated a significant difference in positive mode, as shown in Figure 6c. An apparent separation between them in negative mode is presented in Figure 6d, and samples in the two groups are marked in red and black, respectively. With the results of PN and PN&MA given above, it can be concluded that the two types of samples can be discriminated with 100% accuracy. A total of 21 differential metabolites with statistically significant changes were discovered after analysis. The results of the permutation test with number of running time equal to 200 proved that the model was robust and reliable, as shown in Figure 6e,f. The specificity and sensitivity of the ROC model evaluation in both positive and negative modes reached 100%. Figure 7 shows the ROC results of all the differential molecules discovered in the PN and PN&MD groups, which are presented in Figure 7a–d with a total of 21 metabolite molecules, as introduced above. All the AUC values were above 0.75, which also indicated the high sensitivity and specificity of specific metabolite molecules for disease recognition.

### 3.3. Study of Common Differential Molecular Characteristics between PN&MA and PN&MD

**Figure 8:**
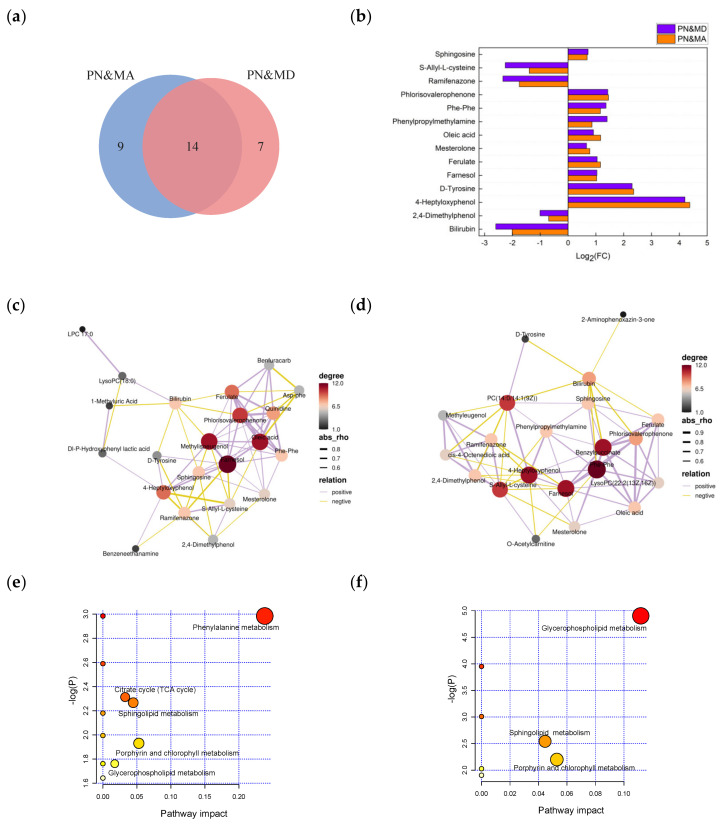
(**a**) A Venn diagram of differential metabolites found in PN and PN&MA groups and PN and PN&MD groups; (**b**) the up–down bar charts of the molecules found in (**a**); (**c**,**d**) the correlation network of differential molecules found in PN and PN&MA groups and PN and PN&MD groups; (**e**,**f**) the findings after metabolic pathway enrichment by using the differential molecules introduced in (**c**,**d**).

A deeper commonality analysis of the differential metabolites found in the PN&MA and PN&MD groups was performed. A total of 14 overlapping differential metabolites in these two groups with the same up- and downregulation trends were recognized. The changes in concentrations of these substances may be strongly correlated with the severity of pneumonia, as shown in Figure 8a,b. Figure 8c,d are the differential metabolite network diagrams of the PN&MA and PN&MD groups. Among the common differential metabolite, farnesol, oleic acid, phlorisovalerophenone, methylisoeugenol, ferulate, 4-Heptyloxyphenol, bilirubin, sphingosine, ramifenazone, and Phe-Phe showed a significant correlation. Figure 8e,f are the enrichment pathway diagrams of differential metabolite molecules of the PN&MA and PN&MD groups, respectively. It can be seen that three common pathways were involved, including porphyrin and chlorophyll metabolism, sphingolipid metabolism, and glycerophospholipid metabolism.

## 4. Discussion

This study identified 14 common differential metabolite molecules and three common pathways in the pneumonia group with metabolic acidosis and myocardial damage compared with the pneumonia group. These findings contribute to a better understanding of the molecular mechanisms underlying pneumonia with metabolic acidosis and myocardial damage. Furthermore, they provide guidance for the identification and validation of biomarkers for pneumonia complications and offer insights into the subsequent clinical application of hierarchical diagnosis and personalized treatment. However, it is important to note that the study has limitations such as the limited number of serum samples and a lack of specific differentiation between various types of pneumonia pathogens. Therefore, further study of large-scale population verification is necessary.

Bilirubin participates in porphyrin and chlorophyll metabolisms. The main source of bilirubin is aging red blood cells. The aging red blood cells are first damaged and transformed into biliverdin with heme from other sources by heme oxygenase. Afterward, biliverdin reacts with biliverdin reductase to give bilirubin, which is involved in porphyrin metabolism in the body [14]. The decrease in bilirubin content in the PN&MD group may be due to the poor function of the mononuclear phagocytic system in newborns. It is possible that aging red blood cells cannot be phagocytosed or that engulfed red blood cells were degraded at a slower rate, resulting in insufficient raw materials for bilirubin production. At the same time, the heme oxygenase activities on the microsomes of mononuclear macrophages reduced the oxidation rate of heme into biliverdin, which also led to a decrease in bilirubin production [15]. In addition, bilirubin itself is a strong antioxidant, and metabolic acidosis and myocardial damage in children may lead to an increase in oxygen free radicals in the body. Part of the bilirubin is consumed to exert its antioxidant function, and the content decreases [16].

Sphingosine, the main component of sphingolipids and a kind of sphingomyelin basic lipid, is involved in sphingolipid metabolism. Sphingomyelin and its metabolites are not only important constituents of cell membranes but also necessary regulators in a variety of signal transduction pathways, which play a significant role in many pathological processes [17,18]. Since sphingomyelin is widely interrelated in the body, its abnormal content will lead to a series of chain reactions leading to inflammation [19]. The content of sphingosine in the two groups of pneumonia complications increased more significantly than that in the PN group, indicating aggravation of an inflammatory reaction.

PC (14:0/14:1 (9Z)) and LysoPC (22:2 (13Z, 16Z)) are involved in glycerophospholipid metabolism. Glycerophospholipids are the most abundant phospholipids in the body and play a role in various physiological functions, such as inflammation and cell damage [20]. In various pathophysiological conditions, the ratio of free and albumin-bound lysophosphatidylcholine (LPC) can be profoundly altered by increasing the production of LPC or lowering plasma albumin levels [21,22], which means that to a certain extent, phospholipids act as important regulators in inflammatory reactions, and the changes in type and content of phospholipids can reflect the severity of inflammation [23]. LPC has many protective or anti-inflammatory effects, and LPC, at a higher level, can act as an anti-inflammatory molecule by producing vascular protective effects through prostacyclin or nitric oxide. During bacterial or viral infections, low levels of LPC can lead to immune disorders [24]. In the present study, the relative contents of substances PC (14:0/14:1 (9Z)) and LysoPC (22:2 (13Z, 16Z)) involved in this pathway increased, indicating that in the PN&MD and PN&MA groups, the inflammatory response was more severe compared with that in PN group. PC (14:0/14:1 (9Z)) and LysoPC (22:2 (13Z, 16Z)) may act as protective or anti-inflammatory compounds.

In addition, some important common differential substances, such as farnesol and oleic acid, have significant specificities, and previous reports have shown that they can regulate inflammatory responses and have beneficial effects on the immune response system in diseases, including edema, allergic asthma, and colon tumors [25]. A series of animal models have demonstrated that farnesol can eliminate tumor growth [26]. It has also been proved that farnesol exhibits potential pro/anti-inflammatory and anticancer effects in various diseases [27]. The increase in farnesol levels in both complication groups indicated that the body was regulating its own exacerbating inflammatory responses. Oleic acid affects cell membrane fluidity, receptors, and intracellular signaling pathways. It can directly regulate the synthesis and activity of antioxidant enzymes and has anti-inflammatory effects, which are reached through inhibiting proinflammatory cytokines and activating anti-inflammatory cytokines [28,29]. As shown in our studies, oleic acid was significantly upregulated in the complication groups, which also reflected the severe inflammatory response in the complication groups. Phe-Phe is a peptide composed of two phenylalanine molecules. Phenylalanine is an essential amino acid and a precursor of D-tyrosine, which can be used for protein synthesis and converted into nonessential amino acid tyrosine [30]. The levels of Phe-Phe and D-tyrosine significantly increased in both complication groups, indicating an abnormal disorder in amino acid metabolism. Changes in amino acid levels can reflect the immune response of the body to infection or tissue damage. Almost all tissue remodeling in the body involves the breakdown and synthesis of proteins, and abnormal amino acid metabolism can have adverse effects on the production of proteins [31]. In a previous study, Phe-Phe was found to be a marker of pancreatic cancer [32]. Therefore, the increase in Phe-Phe concentration may indirectly indicate an increased risk of cancer in the pneumonia complication groups compared with the PN group.

It was evident that pneumonia with metabolic acidosis or myocardial damage was two different types of complications compared with the pneumonia control group, but there were common molecules, and they acted in the same metabolic pathways. The common metabolite molecules revealed that the inflammatory response was more severe in the complication groups, and the risk of cancer tended to increase, while the increased inflammatory response led to a series of ripple reactions, such as abnormal apoptosis of body cells and energy metabolism, and disturbance of amino acid metabolism. These findings of common molecules and metabolic pathways between PN&MA and PN&MD provide a deep understanding of the complications of pneumonia disease, especially for newborns with relatively low immunity.

## 5. Conclusions

In this work, an untargeted UPLC–HRMS-based metabolomics approach was developed and applied for the study of neonatal pneumonia and pneumonia complicated by metabolic acidosis or myocardial damage. Serum samples were collected and analyzed to explore the important differential metabolites between healthy control and disease groups. The molecular mechanisms of actions that disrupt the pathways were discovered. Based on these findings, the common molecular mechanisms of the two complications of pneumonia were investigated. The results showed a significant decrease in bilirubin levels in the PN&MA and PN&MD groups, which implied an increased inflammatory response. Meanwhile, the levels of farnesol and sphingosine as anti-inflammatory substances increased, indicating the presence of the self-regulation of organisms in response to the exacerbation of the inflammatory response. The changes in the levels of these common substances and the metabolic pathways involved were mainly closely related to the series of immune responses caused by the exacerbation of inflammation levels. Comparing groups PN and PN&MA and PN and PN&MD, the relative content of common substances showed the same upward and downward trends with varying degrees, but the differences were not significant. The changes in the contents of these substances may represent changes in the severity of pneumonia complications in newborns. In the future, a targeted study will be conducted to take the effects of multiple pathologies into consideration for validation and clinical application, which aims to fully validate the investigation of the potential programming mechanisms of these differential molecules, as well as the effects and mechanisms of repair after drug treatment and/or prognostic assessment.

## Figures and Tables

**Table 1:** Each IS and its corresponding concentration in the prepared mixed standard solution.

No.	Compound Name	Concentration (μg/mL)
1	choline-d4	2.0
2	cannitine C2:0-d3	0.16
3	phenylalanine-d5	3.5
4	FFA16:0-d3	2.5
5	FFA18:0-d3	2.5
6	cannitine C10:0-d3	0.1
7	cannitine C8:0-d4	0.1
8	CA-d4	1.85
9	CDCA-d4	1.5
10	cannitine C16:0-d3	0.15
11	LPC 19:0	0.75
12	SM d30:1	0.75
13	glutamic acid-d3	0.15

## Data Availability

The data presented in this study are available on request from the corresponding author. Data is not publicly available due to privacy.

## Supplementary Materials

The following supporting information can be downloaded at: https://www.mdpi.com/article/10.3390/metabo13111118/s1. Table S1: diseases abbreviations; Table S2: annotation results.
